# Contrasting diets reveal metabolic plasticity in the tree-killing beetle, *Anoplophora glabripennis* (Cerambycidae: Lamiinae)

**DOI:** 10.1038/srep33813

**Published:** 2016-09-22

**Authors:** Charles J. Mason, Erin D. Scully, Scott M. Geib, Kelli Hoover

**Affiliations:** 1 Department of Entomology and Center for Chemical Ecology, The Pennsylvania State University, University Park, PA 16802 USA; 2 Stored Product Insect and Engineering Research Unit, USDA, Agricultural Research Service, Center for Grain and Animal Health Research, Manhattan, KS 66502 USA; 3 Tropical Crop and Commodity Protection Research Unit, USDA, Agricultural Research Service, Daniel K. Inouye Pacific Basin Agricultural Research Center, Hilo, HI 96720 USA

## Abstract

Wood-feeding insects encounter challenging diets containing low protein quantities, recalcitrant carbohydrate sources, and plant defensive compounds. The Asian longhorned beetle (*Anoplophora glabripennis*) is a wood-feeding insect that attacks and kills a diversity of hardwood tree species. We compared gene expression of midguts collected from larvae feeding in a preferred tree, sugar maple, to those consuming a nutrient-rich artificial diet, to identify genes putatively involved in host plant utilization. *Anoplophora glabripennis* larvae exhibited differential expression of ~3600 genes in response to different diets. Genes with predicted capacity for plant and microbial carbohydrate usage, detoxification, nutrient recycling, and immune-related genes relevant for facilitating interactions with microbial symbionts were upregulated in wood-feeding larvae compared to larvae feeding in artificial diet. Upregulation of genes involved in protein degradation and synthesis was also observed, suggesting that proteins incur more rapid turnover in insects consuming wood. Additionally, wood-feeding individuals exhibited elevated expression of several mitochondrial cytochrome C oxidase genes, suggesting increased aerobic respiration compared to diet-fed larvae. These results indicate that *A. glabripennis* modulates digestive and basal gene expression when larvae are feeding in a nutrient-poor, yet suitable host plant compared to a tractable and nutrient-rich diet that is free of plant defensive compounds.

Insect herbivores encounter a diversity of food substrates that vary extensively in their nutritive qualities and secondary metabolite composition^1^,^2^. Insects feeding beneath the bark in the subcortical tissues of trees encounter a particularly challenging environment. Woody tissues have very low quantities of nitrogen^3^,^4^, are comprised predominantly of poorly accessible sources of carbohydrates in the form of lignocellulose^5^, and can be heavily defended with phytochemicals. Xylophagous (wood-feeding) insects have adapted multiple, complementary mechanisms to contend with these challenges. Among these adaptations are host selection behaviors, associations with microbial symbionts, and intrinsic digestive and detoxification capabilities.

Some xylophagous insects have suites of genes that enable them to consume living host plants. For instance, transcriptome and genome sequencing of xylophagous beetles have revealed expansions of several families of digestive glycoside hydrolases, detoxification genes, and digestive proteinases^6^,^7^,^8^,^9^. Cerambycid beetles, like other herbivorous insects, also encode genes that enable them to cope with nutrient poor conditions^5^,^6^,^10^,^11^,^12^,^13^,^14^,^15^,^16^. However, differential expression of digestive and detoxification genes to accommodate different food substrates is not well understood in most herbivorous insects, especially those that feed on wood.

In addition to insects’ intrinsic digestive capabilities, many wood-feeding herbivores are also associated with microbes, which likely contribute to digestive processes. Microbial symbionts carried externally can be introduced into trees and directly consumed in lieu of wood, or be housed in the herbivore’s gut tissues to help facilitate digestive processes. Microbes associated with wood-feeding insects can help metabolize components of lignocellulose to liberate more accessible carbohydrates^17^,^18^, fix or recycle nitrogen^12^,^19^, and provide potential sources of sterols^20^,^21^, and other important vitamins and nutrients. Moreover, microbes can metabolize various classes of tree phytochemical defensive compounds to alleviate toxicity to their herbivore hosts^22^,^23^,^24^,^25^.

The Asian longhorned beetle, *Anoplophora glabripennis* (Cerambycidae: Lamiinae), is a polyphagous pest of hardwood trees that colonizes both stressed and healthy living trees. *Anoplophora glabripennis* is native to eastern Asia, but has established several invasive breeding populations in the United States and Europe. In the United States, maples (*Acer* spp.) are most frequently attacked and killed by *A. glabripennis*^26^,^27^. While adult beetles perform multiple ovipositions into a single tree, the larvae feed solitarily. Larvae have a long, serpentine gut system, consisting primarily of midgut with a short hindgut (Supplemental Fig. 1). The *A. glabripennis* larval midgut is populated with a diverse assemblage of bacteria and yeasts^7^,^14^,^28^, and filamentous fungus belonging to the *Fusarium solani* species complex 6 mating population (FSSC6)^29^,^30^,^31^. Bacterial and fungal symbionts have the predicted enzymatic capacity to digest recalcitrant lignin, cellulose, hemicellulose, and pectin^16^,^28^ and are responsible for essential amino acid provisioning^28^,^30^,^32^. Metatranscriptome analysis of the larval midgut also revealed that the microbiome expresses genes that complement and augment the beetle’s endogenous digestive and nutrient acquiring capacities^7^. Along with these associates, *A. glabripennis* produces a number of endogenous carbohydrase enzymes, detoxification enzymes, and digestive serine proteinases, which work in tandem with microbial enzymes to facilitate digestion of woody tissues and nutrient acquisition^28^.

*Anoplophora glabripennis* represents an illuminating model for investigating how wood colonizing insects cope with nutritionally deficient, chemically defended dietary substrates^33^. This beetle is amenable to rearing in living trees, recently cut bolts of wood and on an artificial diet^34^. In this study, we analyzed endogenous midgut gene expression of larval *A. glabripennis* using global transcriptome analysis, comparing larvae reared in trees to larvae reared on a nutrient-rich dietary substrate. For this study, we focused on identifying differences in expression levels in genes related to digestion, detoxification, and microbial interactions as well as basal metabolic processes.

## Results

### Overview of Comparative RNA-Seq Analysis

**Figure 1:**
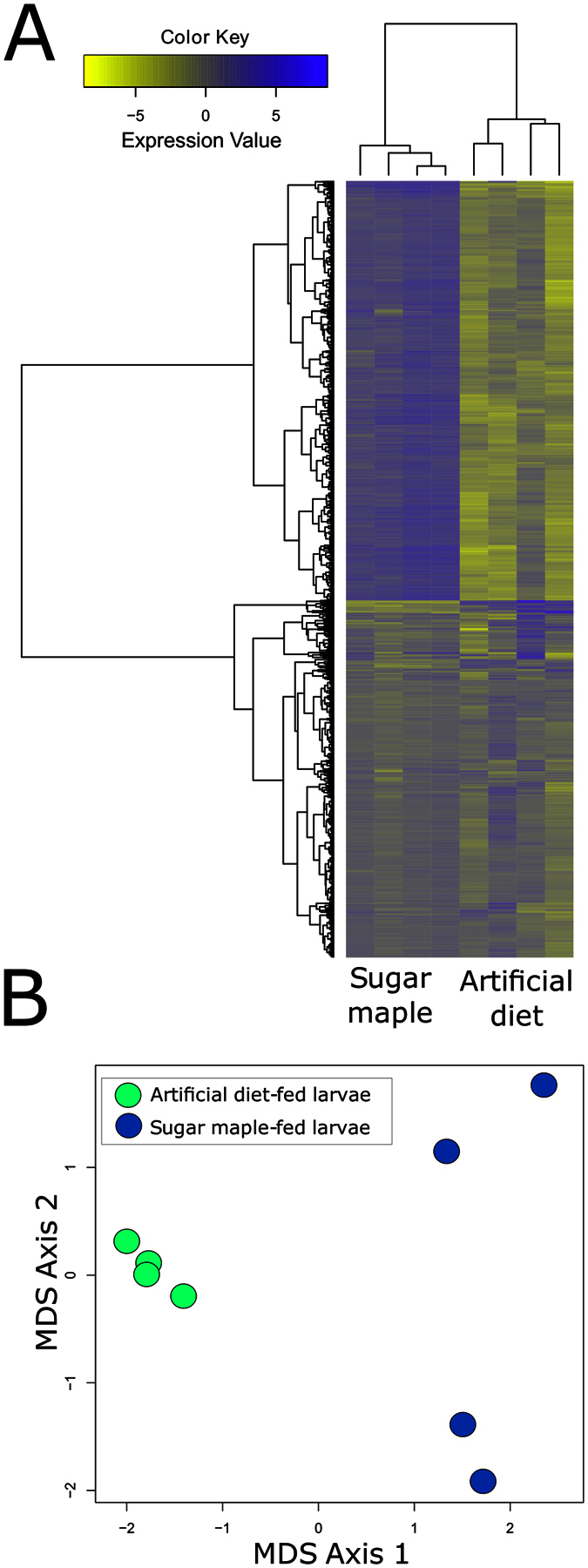
Heatmap analysis of differentially expressed genes identified in *A. glabripennis* larvae feeding in sugar maple and artificial diet (**A**) and MDS analysis of expression patterns of larvae feeding in artificial diet and sugar maple (**B**). Genes with ≥1.25 or ≤−1.25 log fold changes and FDR adjusted p-values ≤ 0.05 were identified as being differentially expressed. Genes were clustered using Ward’s method and the heatmap was created using ‘heatmap2’ in the R statistical computing environment. Blue indicates higher expression levels; yellow indicates lower expression levels.

Over 8,000 expressed genes (>10 mapped reads) comprising ~36% of the protein coding genes in the *A. glabripennis* genome were detected in the midguts of actively feeding larvae. (Supplemental Data 1). Expression of ~3600 of these genes were significantly impacted by feeding substrate (Fig. 1A), using a log-fold change cutoff of ± 1.25 and a false discovery rate (FDR) corrected p-value ≤ 0.05. A less conservative filtering approach with no log-fold change cutoff identified only 300 additional differential expressed genes (Supplemental Data 1). Nonmetric multidimensional scaling (MDS) analysis of the normalized reads indicated clear separation between the insects consuming the two different diets (Fig. 1B). Expression profiles of midguts collected from artificial diet-fed insects were more similar to one another and were less variable than those of larvae consuming sugar maple (wood) as depicted in the separation of two groups of sugar maple-reared insects along MDS-2. Approximately 100 genes could explain a combined total of 63% of the variance along the MDS-2 axis (Supplemental Data 2). The heterogeneous nature of the sugar maple wood in terms of polysaccharide composition and nutritional quality was reflected in our findings in that several of the genes with variable expression levels between the two groups of midguts collected from sugar maple-reared insects had putative roles in digestion and nutrient acquisition, which included carboxylesterase (AGLA013185), aldehyde dehydrogenase (AGLA009921), nucleoside transporter (AGLA007856), sugar transporter (AGLA00783), subtilase serine proteinase (AGLA004564), GH 79 (AGLA003075), trypsin (AGLA002776), and aldo-keto reductase (AGLA000966). Additionally, the transcriptome profiles of sugar maple reared insects also differed in the expression level of 33 hypothetical proteins and two uncharacterized proteins. The full list of the top 100 genes contributing to variance along MDS-2 is located in Supplemental Data 2.

**Figure 2:**
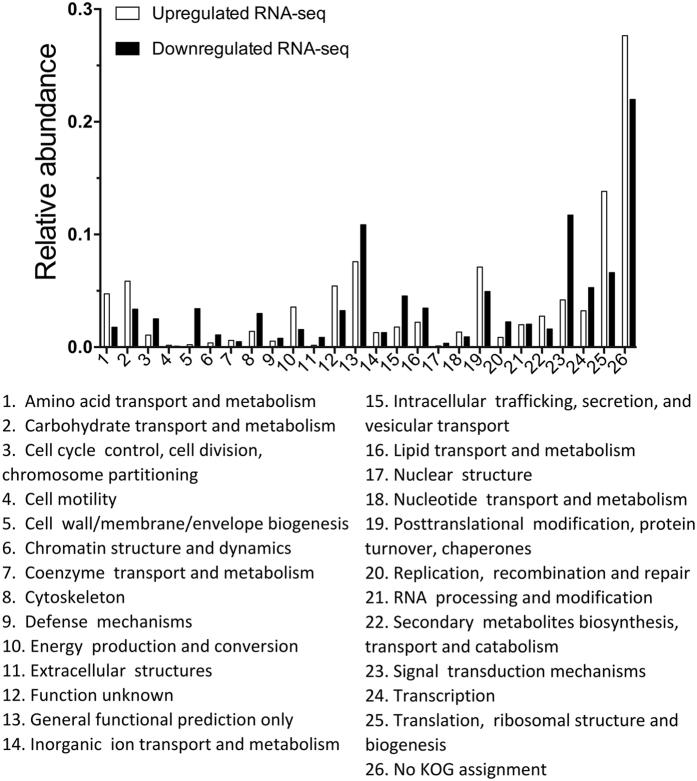
Abundances of clusters of orthologous (KOG) categories in genes more highly expressed in sugar maple- and diet- fed larvae. Differentially expressed genes in the midguts of larvae fed in sugar maple and artificial diet were assigned to KOG categories using RPS BLAST. The e-value cutoff was 0.00001 and genes were assigned to the KOG category of their top scoring BLAST match.

Using a conservative log-fold change of ±1.25 and an FDR corrected P-value ≤ 0.05, 1,661 genes involved in digestion, detoxification of phytochemicals, stress response, nutrient recycling and scavenging, and interactions with midgut microbes were upregulated in wood-fed larvae compared to larvae fed on artificial diet. The majority of these upregulated genes were assigned to the following KOG categories: “amino acid transport and metabolism” (4.8% of the upregulated genes); “carbohydrate transport and metabolism” (5.9%); “energy production and conversion” (3.6%); “secondary metabolites biosynthesis, transport, and catabolism” (2.7%); “posttranslational modification, protein turnover, chaperones and translation” (2.9%); and “ribosomal structure and biogenesis” (7.2%) (Fig. 2). In wood-fed insects, we observed enrichment of 30 biological process and 55 molecular function GO terms, including “hydrolysis of O-glycosyl and ester linkages”, “proteolysis”, “oxidoreductase activity”, “polygalacturonase activity”, “antioxidant activity”, and “structural constituents of ribosomes” (Supplemental Table 1).

1,952 genes were downregulated in *A. glabripennis* larvae that consumed wood compared to larvae fed on artificial diet. Eukaryotic clusters of orthologous gene (KOG) categories included: “cell wall/ membrane/ envelope biogenesis” (3.4% of the downregulated genes); “cytoskeleton, intracellular trafficking, secretion, and vesicular transport” (3.0%); “replication, recombination and repair” (2.3%); and “signal transduction mechanisms” (11.8%) (Fig. 2). We identified eight biological process and 58 molecular function terms that were enriched in insects feeding in artificial diet compared to sugar maple using Gene Ontology (GO) enrichment analysis (Supplemental Table 2). The enriched terms included categories of genes involved in signal transduction, cuticle biosynthesis, ion binding, and nucleic acid binding.

### Carbohydrate Metabolism: Genes Involved in the Metabolism of Starch, Sucrose, and α-1,3 and α-1,6 Glycosides were Induced in Sugar Maple-Reared Insects

Insect-derived cellulases, β-glucosidases, xylanases, and polygalacturonases involved in the digestion of plant primary and secondary cell wall polysaccharides in *A. glabripennis* and other cerambycids have been identified and discussed elsewhere^6^,^9^. While these genes were also upregulated in wood-fed larvae^33^, here we focus on how the insect obtains sugars from other carbohydrate sources.

**Figure 3:**
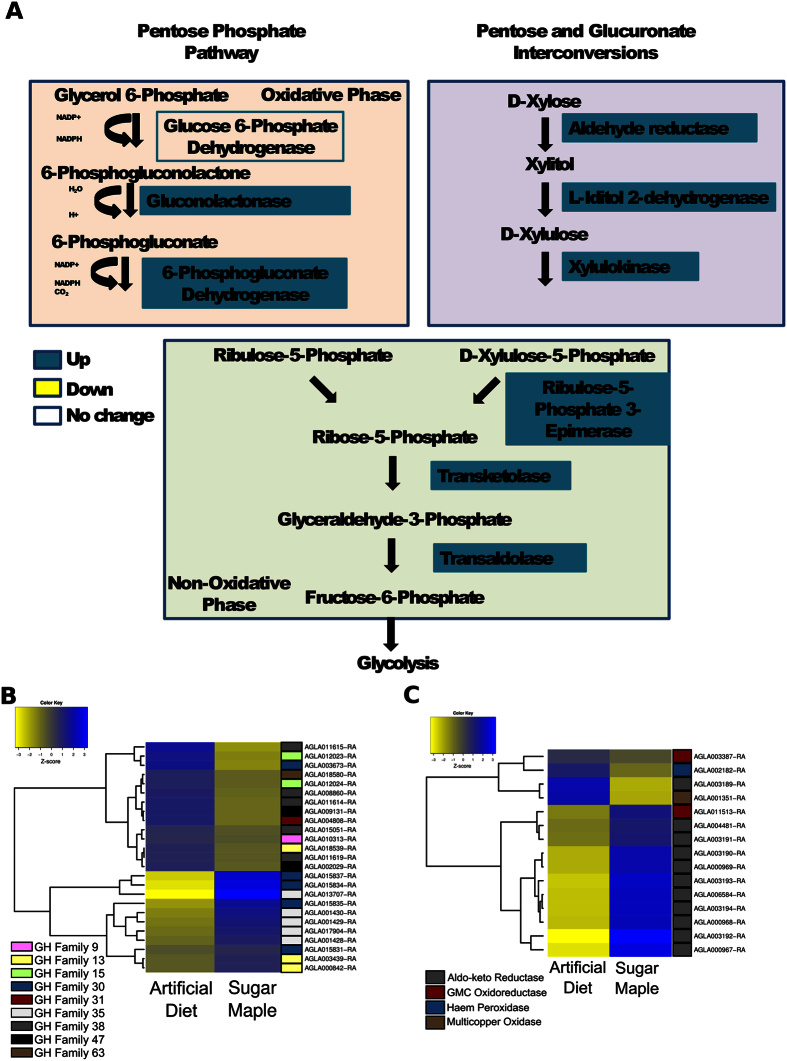
Expression patterns of genes related to carbohydrate digestion. (**A**) Pentose phosphate pathway. The pentose phosphate pathway was induced in larvae feeding in sugar maple. Genes highlighted in blue were more highly induced in larvae feeding in sugar maple while genes highlighted in yellow were more highly induced in larvae feeding in artificial diet. (**B**) Expression patterns of glycoside hydrolases detected in larval midguts. Several genes with the ability to metabolize non-plant cell wall carbohydrates were induced in larvae fed in either sugar maple or artificial diet. Blue indicates high expression levels and yellow indicates low expression levels. (**C**) Expression patterns of other digestive related genes detected in larval midguts. Aldo-keto reductases, GMC oxidoreductases, and heme peroxidases, have been previously hypothesized to be involved in woody tissue degradation in wood-feeding insects. Blue indicates high expression levels; yellow indicates low expression levels.

The entire pentose phosphate pathway was upregulated in larvae that consumed wood compared to those fed in artificial diet (Fig. 3A). Enzymes responsible for catalyzing pentose and glucoronate interconversions, which can convert pentose sugars found in hemicellulose (i.e., xylose and arabinose) into substrates that can be used for glycolysis, were upregulated in wood-fed larvae. Four genes with products predicted to hydrolyze α-1,4-linked glucose found in amylose and other starches exhibited higher expression in these larvae (Fig. 3B). A single gene annotated as invertase, which is responsible for catalyzing the conversion of sucrose to glucose, was also more highly expressed in wood-feeding larvae (Fig. 3B). The *A. glabripennis* genome encodes 30 glucose-methanol-choline (GMC) oxidoreductases, 25 aldo-keto reductase genes (AKRs), 12 animal heme peroxidases, and three laccases/multicopper oxidases. Of these, larvae feeding in sugar maple expressed more transcripts of 10 AKR and two GMC oxidoreductase genes than diet fed larvae (Fig. 3C). In contrast, no peroxidases or laccases were upregulated in the midguts of sugar maple-fed larvae.

Several digestive-related genes were downregulated in wood-fed larvae compared to diet-fed larvae. The glucose hydrolase (GH) families that were expressed at lower levels in larvae consuming wood included: four GH family 1 genes with amino acid similarity to myrosinases and β-glucosidases, two GH family 15 family genes (with blastp matches to exo-acting α-glucosidases), five GH 18 genes (chitinases), one GH family 20 gene (hexosamidase), one GH 30 gene (glucosylceramidase), one GH 31 gene (α-glucosidase), seven GH 38 genes (α-mannosidases), and two GH family 47 genes (likely involved in processing 1,2-linked α-D-mannose sugars in asparagine-linked mannose oligosaccharides) (Fig. 3B). Two AKR genes, one laccase, three GMC oxidoreductases, and one animal heme peroxidase were more highly expressed in diet-fed compared with wood-fed larvae (Fig. 3C).

### Genes Involved in Amelioration of Oxidative Stressed are Highly Induced in Sugar Maple Reared Insects

**Figure 4:**
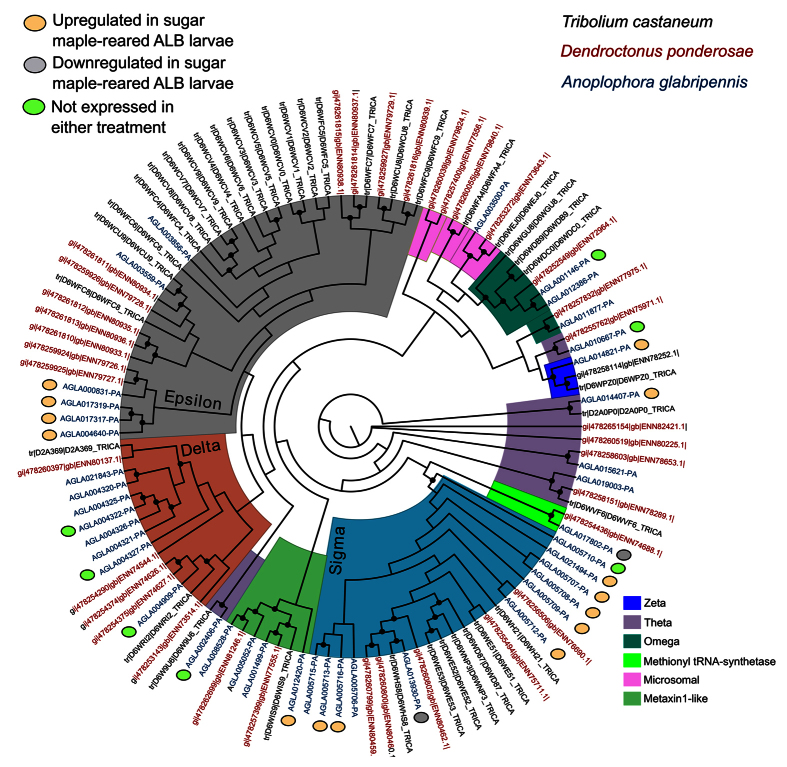
Maximum likelihood analysis of glutathione S-transferases (GSTs) detected in the *A. glabripennis* genome compared to other wood-feeding colepoterans. A maximum likelihood analysis of GSTs detected in the *A. glabripennis* (ALB), mountain pine beetle (MPB), and *Tribolium castaneum* (red flower beetle) genomes was performed using the program Garli. The LG + I evolutionary model was selected as the optimal rate matrix using ProtTest and 500 bootstrap pseudoreplicates were analyzed. The genes were assigned to clades based on relatedness to red flour beetle and mountain pine beetle GSTs, which had been classified previously^8^. Brown circles denote GSTs that were induced by feeding in sugar maple, while grey circles denote GSTs that were more strongly induced in the diet-fed larvae.

The expression of cytochrome P450s, carboxylesterases, and UDP-glucuronosyl transferases in *A. glabripennis* larvae have been discussed previously^6^. Genes encoding these enzymes were upregulated in wood-fed larvae^33^. Several other pathways and genes were activated in sugar maple reared insects with putative involvement in detoxification. Twelve glutathione S-transferases (GSTs) were upregulated in wood-fed larvae (Fig. 4). The most prominent GST families encoded by *A. glabripennis* included the delta (eight genes), epsilon (six genes), and sigma (12 genes) families, which were mostly found arranged in tandem arrays on various genomic scaffolds. Genes belonging to the zeta, theta, omega, and metaxin-like families were also found in the genome, but predominantly occurred as singletons. Seven tandemly duplicated sigma family genes were induced in wood-fed larvae (Fig. 4). Other upregulated GSTs included four epsilon family genes, two theta family genes, and three sigma family genes found as singletons. In contrast, only two GST genes were downregulated in wood-fed larvae, while seven were not expressed in the midguts from insects feeding in either substrate.

Genes and pathways involved in response to oxidative stress were significantly upregulated in wood-fed larvae compared with those fed artificial diet. This included the entire taurine, ubiquinone, and glutathione biosynthesis pathways responsible for synthesizing their respective antioxidants. Other upregulated genes in wood-fed larvae with putative antioxidant ability included superoxide dismutase and thioredoxin reductase I.

The two diets influenced expression levels of several genes with putative detoxification capacities. Rhodanese genes involved in mitochondrial cyanide detoxification, as well as genes putatively involved in dehydrogenating alcohols and aldehydes, were upregulated in wood-fed larvae compared with larvae fed artificial diet. Twenty short chain dehydrogenases were also induced in larvae feeding in sugar maple. In contrast, only seven short chain reductases, two alcohol dehydrogenases, and a single aldehyde dehydrogenase were more highly expressed in insects feeding in artificial diet.

Several detoxification genes were more highly expressed in artificial diet-fed insects than in wood-fed larvae; these included 11 carboxylesterases, 10 cytochrome P450s, 11 UDP-glucuronsyl transferases, and two GSTs (Supplemental Table 3). While these genes have proposed roles in digestive physiology, their upregulation in the guts of insects feeding in artificial diet suggests that they could serve other physiological roles, such as the detoxification and elimination of endogenous waste products.

**Figure 5:**
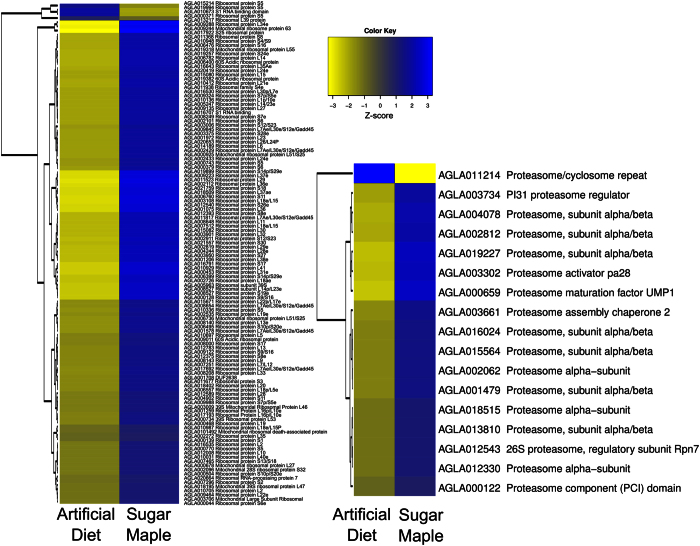
Expression levels of genes related to protein synthesis and recycling. The expression levels of genes related to protein synthesis, ribosome biogenesis, proteolysis, and recycling were significantly upregulated in midguts collected from larvae feeding in sugar maple. The z-score heatmap was created using the ‘heatmap2’ package in R. Blue signifies high expression levels, while yellow signifies low expression levels.

Pathways involved in protein degradation and protein biosynthesis, which can be activated under periods of stress, were significantly upregulated in wood-fed larvae (Fig. 5). In particular, we observed higher expression of genes and proteins involved in ubiquitination and proteasomal degradation. Genes involved in translation and protein synthesis were also upregulated in wood-fed larvae (Fig. 5). Several genes encoding heat shock family 20 and 70 proteins, possibly serving as chaperones to damaged proteins (1.1–3.5 fold) were upregulated in insects consuming wood (Supplemental Table 4).

### Upregulation of Genes Involved in Nutrient Assimilation and Scavenging

**Figure 6:**
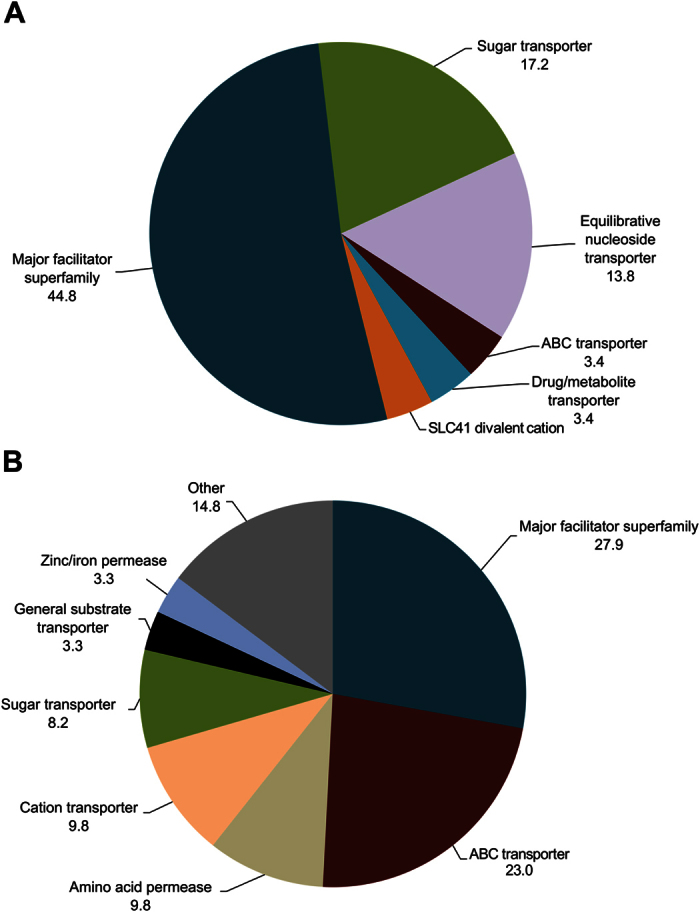
Expression levels of transporters expressed in the midgut. The expression levels of several transporters with potential roles in nutrient assimilation were impacted by the feeding substrate.

Compared to artificial diet, wood-fed larvae exhibited greater expression of several genes and pathways involved in salvaging amino acids and nucleotides. For example, genes involved in salvage of the non-essential amino acids methionine (acireductone dioxygenase) and cysteine (cysteine oxygenase), ribose sugar salvage (5′-nucleotidase), and purine salvage (AMP deaminase) were more highly expressed in beetles feeding in wood. Genes encoding enzymes for metabolizing essential (tyrosine and tryptophan) and non-essential (serine and threonine) amino acids, as well as genes that encode enzymes that catalyze deamination reactions, such as aminohydrolase, amine oxidase, and asparaginase/glutaminase were upregulated in sugar maple-fed larvae. Likewise, genes encoding transporters responsible for assimilating nutrients from the environment were also strongly impacted by diet (Fig. 6). Major facilitator transporters represented the majority of the transporters whose expression levels were upregulated in both diets. In sugar maple reared insects, expression level of several equilibrative nucleoside transporters and sugar transporters were highly upregulated while ABC transporters and amino acid permeases represented major classes of transporters upregulated in the artificial diet-reared insects.

Genes involved in aerobic respiration and ATP production were upregulated in wood-fed larvae compared with artificial diet. For example, when feeding in wood, 13 mitochondrial cytochrome C oxidase (COX) protein genes were induced. These included COX subunits Ib, II, III, IV, Va, Vb, ViA, ViC, VIIc (upregulated at levels ranging from 1.25-fold to 3.63-fold) as well as three COX assembly proteins (1.6-fold to 2.3-fold) and one COX biogenesis protein (2.2-fold). We also observed upregulation of six mitochondrial ATP synthase genes (2.0 to 2.1-fold) and one mitochondrial gene (1.25-fold) in wood-fed larvae. In contrast, larvae feeding in artificial diet only expressed eight mitochondrial carrier proteins (1.25 to 3.1-fold) at higher levels compared to those reared in sugar maple; no cytochrome C oxidase subunits were more highly expressed.

### Genes Involved in Interactions with Gut Microbes Highly Expressed in Larvae Consuming Sugar Maple

**Figure 7:**
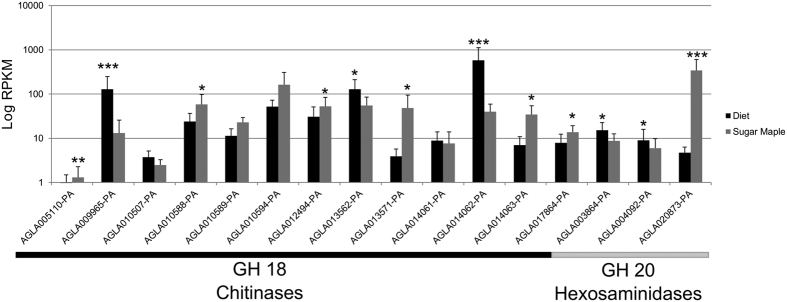
Expression levels of GH 18 chitinases and GH 20 hexosamidases in the midgut. Genes encoding enzymes with putative roles in the breakdown of the N-acetylglucosamine polymer, chitin, were impacted by the feeding substrate. RPKM values were computed using the ‘rpkm’ function in edgeR. *p < 0.05; **p < 0.01, **p < 0.001.

Several genes were upregulated in wood-fed larvae that potentially facilitate gut microbial interactions. For example, genes with predicted involvement in microbial sugar metabolism and pathogen response were upregulated. Also upregulated were genes encoding β-1,3 glucan binding proteins, chitinases and genes assigned to the glucosaminoglycan degradation pathway, suggesting that fungal chitin could serve as a potential source of carbon (Fig. 7). In contrast, there was no differences in expression between the two treatments in genes involved in chitin biosynthesis, such as chitin synthase and peritrophins, indicating that the higher expression levels of chitinases by wood-fed insects is unlikely related to remodeling of the peritrophic matrix (PM). Further, four of these genes lacked a C-terminal cysteine-rich chitin-binding domain (CBD), which mediates binding to the PM^35^. These four chitinases (AGLA014063, AGLA010588, AGLA014061, and AGLA014062) could possibly serve roles in digestion of fungal chitin or chitin derived from other dietary sources.

Trehalase genes containing signal peptides were upregulated in wood-fed larvae, suggesting that these proteins are secreted into the extracellular environment. These encoded proteins are likely involved in metabolizing exogenous sources of trehalose as described previously for soluble trehalases^36^. Trehalose is a principle carbohydrate present in the hemolymph and a building block for chitin. Additionally, trehalose is a storage compound in several microbial taxa and is produced by plants during stress responses; therefore, the elevated expression levels of soluble trehalases could be related to metabolism of either microbial- or plant-derived sugars in the midgut.

A substantial number of genes that encode pathogen-responsive related elements had elevated expression levels in the midguts of wood-fed larvae. Overall, some of the most strongly upregulated genes in larvae feeding in wood included those encoding three lectin C-type domain proteins (AGLA006632: 4.97-fold; AGLA006633: 4.70-fold; AGLA008786: 4.57-fold). These proteins contain carbohydrate-binding domains putatively involved in immune responses. Larvae consuming wood also had higher expression of genes encoding allergen domain proteins involved in pattern recognition and interactions with microbes. Wood-fed larvae had higher expression of genes with putative roles in microbial interactions, including a small defensive gene (arthropod defensin) (AGLA002833: 4.59-fold) and two attacin genes whose products are capable of binding to bacterial lipopolysaccharides (AGLA003080: 5.20-fold; AGLA003081: 6.34-fold). Other upregulated genes included five leucine repeat rich (LRR) genes, one thaumatin domain gene, one I-set domain protein, and two coleoptericins. Genes that encode enzymatic and structural components of lysozomes for phagocytosis were also upregulated in wood-fed larvae.

Several genes involved in microbial interactions were more highly expressed in the diet treatment. Twelve LRR genes with putative involvement in microbial pattern recognition, five I-set domain genes, and one peptidoglycan binding gene were upregulated in larvae fed artificial diet. Additionally, one serine proteinase inhibitor and one trypsin inhibitor that could serve roles in microbial interactions were induced in the diet-fed larvae (Supplemental Fig. 2).

## Discussion

Given that plant hosts often have varying nutritional content and defensive capabilities, the ability of polyphagous insect herbivores to modulate their digestion to differing diets is important. Among insect herbivore feeding guilds, xylophagy poses significant nutritional challenges. In our study, we identified marked differences in midgut gene expression in *A. glabripennis* midgut larvae in their digestive and basal metabolic processes between larvae fed in wood or on artificial diet. In general, genes involved in accessing nutrients either directly from wood or indirectly from gut microbiota were upregulated in wood-feeding larvae. Large numbers of genes encoding putative detoxification enzymes were also induced in the midguts of beetles feeding in sugar maple. Additionally, expression of genes encoding enzymes involved in protein degradation and biosynthesis were elevated in insects consuming wood. These differences indicate that expression of digestive and detoxification genes can exhibit considerable plasticity. *Anoplophora glabripennis* feeding in wood appear to acquire sugars and amino acids from a more diverse set of substrates, undergo greater turnover of proteins, and have elevated levels of aerobic respiration, as suggested by the upregulation of mitochondrial COX genes.

Ingestion of toxins and exposure to reactive oxygen species (ROS) produced by host plants in response to herbivory can induce oxidative stress in the gut, which in turn causes significant damage to structural and secretive digestive midgut proteins^37^. In this system, phenolics constitutively present in sugar maple phloem may form agylcone and quinone free radicals, damaging *A. glabripennis* gut tissues. Additionally, lignin degradation, a process occurring in the gut of *A. glabripennis*^18^, is accompanied by the formation of free radicals and the release of phenolic compounds from the lignin polymer. In insects feeding in wood, we observed upregulation of genes with products likely serving to overcome stress associated with production of ROS and responses to plant defenses. For example, insects feeding in wood had higher expression of genes involved in ubiquitination and proteasomal degradation and protein biosynthesis, which serve to eliminate and then replace damaged proteins. Further, expression levels of several classes of detoxification genes (GSTs, short chain dehydrogenases, and alcohol/aldehyde dehydrogenases) and antioxidants (glutathione, taurine, and ubiquinone) were elevated when larvae consumed wood.

Despite the large number of upregulated detoxification-related genes in wood-fed larvae, some genes were more highly expressed in larvae fed in artificial diet. The artificial diet for *A. glabripennis* contains antimicrobial compounds to reduce spoilage^34^. High concentrations of methylparaben, for example, have been documented to reduce herbivore fitness parameters in other insect species^38^. In contrast, the many genes responsible for mitigating oxidative stress in other animals were downregulated in larvae fed artificial diets^39^, which is likely due to the presence of less recalcitrant sources of carbohydrates, and the absence of lignin and plant defensive compounds.

Feeding in woody tissues is presumably an energetically demanding process. We observed induction of genes involved in oxidative phosphorylation in wood-feeding larvae, including 13 mitochondrial COX genes, which is indicative of increased levels of aerobic respiration. While this may be related to increased protein turnover, there are other aspects of consuming wood that may also affect respiration levels. Compared to artificial diet, wood has lower nutrient content and contains recalcitrant sources of carbohydrates and defense metabolites. Lignocellulose digestion and allelochemical detoxification are both energetically demanding and can increase respiration and ATP consumption^40^,^41^.

Our results indicate that *A. glabripennis* uses a diversity of sugar and nutrient sources that arise from both the plant and microbiota. For example, starches and amylose (possibly derived from host plants) appear to be utilized due to the expression of multiple genes with products predicted to hydrolyze alpha-1,4 linked glucoses. Sucrose, a major photosynthetic product transported through the phloem, also appears to be utilized based on the elevated expression of invertase. The induction of the pentose glucuronate interconversion pathway in insects feeding in sugar maple may also allow *A. glabripennis* larvae to metabolize pentose sugars (e.g., arabinose and xylose) liberated from hemicellulose. Previously, it was hypothesized that microbial symbionts inhabiting the midgut convert sugars released from hemicellulose into pyruvate, a key metabolic intermediate in several metabolic pathways (e.g., glycolysis and fatty acid biosynthesis) based on the high expression levels of xylose isomerase and xylose reductase genes derived from gut microbes^7^. While it is likely that gut microbes metabolize some of the sugars released from hemicelluloses, the elevated expression levels of the pentose glucuronate pathway suggests that the beetle itself may also be able to metabolize these sugars to produce ATP, fatty acids, and non-essential amino acids. Midgut bacteria and fungi have previously been implicated in provisioning essential amino acids in this species^7^,^32^,^42^, and our results suggest they are also involved in provisioning carbohydrates. Expression of genes encoding chitinases, ceramidases, and trehalases, all whose respective carbohydrate molecules can be associated with microbial cell walls, were upregulated in wood-fed insects. Genes encoding proteins with putative scavenging abilities support previous suggestions of microbiota provisioning a number of important nutrients.

There was a significant upregulation of immune-related genes in *A. glabripennis* larvae consuming wood. The importance of immune genes in the interaction and maintenance of symbionts has been suggested elsewhere^43^,^44^, and the upregulation of these genes in wood-fed larvae likely facilitates interactions with microbial partners. In contrast to sugar maple, there was lower expression of many gut immune genes when *A. glabripennis* larvae consumed artificial diet. These differences may be due to several non-exclusive mechanisms. First, the artificial diet is a complete source of high quality protein and carbohydrates, and thus the larvae may not need contributions from gut symbionts for growth and development. Second, the antimicrobials included in the diet significantly reduce bacterial and fungal growth in culture (C. Mason, unpublished data) and have direct effects on the community by reducing overall abundance of microbial taxa in the gut. Third, dramatic shifts in bacterial assemblages have been documented when *A. glabripennis* feeds on artificial diet^14^.

There are several aspects of *A. glabripennis’* responses to feeding in different diets that diverge from similar observations in other wood-feeding insects. For example, glutathione S-transferase (GST) genes in the *A. glabripennis* genome were assigned to nine families, all of which have been previously identified in the genomes of other beetle species. However, unlike *Tribolium castaneum* or *Dendroctonus ponderosae*, which are both dominated by glutathione S-transferases assigned to the epsilon family, the most prominent GST gene families in the *A. glabripennis* genome were the delta, epsilon, and sigma families^8^,^45^. Another contrast was laccase expression in the midgut. In xylophagous termites, laccases have been shown to oxidize monolignols and other dietary phenolics^46^. While several laccases are encoded by the *A. glabripennis* genome, differential laccase expression was not observed in this study. This finding supports previous studies suggesting that insect-expressed laccases do not significantly contribute to lignin degradation in *A. glabripennis*^6^,^7^, but could be accomplished by the *Fusarium solani* (FSSC6) symbiont or other gut microbes using enzymes yet to be identified^16^.

Despite the marked differences in gene expression detected between *A. glabripennis* feeding in artificial diet and wood, we observed no differences in genes that might contribute to peritrophic matrix remodeling. These included midgut chitin synthases and peritrophins. This suggests that the peritrophic matrix is one of the components more resistant to changes in gene expression in larvae fed on the two diets used in this study. This could be due to the fact that both diets are suitable for growth and development by *A. glabripennis* larvae, and it is possible that less suitable hosts may alter peritrophic matrix integrity and remodeling.

The midgut transcriptomes of wood- and artificial diet-fed larvae provided stark contrasts in physiological processes involved in digestion and maintaining symbioses. Collectively, these results show that *A. glabripennis* larvae exhibit considerable plasticity related not only to their digestive physiology, but also to basal intracellular processes, such as sugar metabolism, protein biosynthesis and turnover, ATP production, and aerobic respiration. There are several areas of future research that can expand upon these findings. *Anoplophora glabripennis* feeds in a number of hosts, and comparing expression patterns among larvae fed in different host plants would improve our understanding of how the larvae modify digestion in response to variations in nutritional quality and plant defenses. Additionally, *A. glabripennis* feeds on dramatically different substrates throughout its development (adults feed on bark and petioles; larvae feed on phloem then move into heartwood); ontogenetic comparisons would provide valuable insights into digestive plasticity. Finally, manipulating artificial diet nutritional content and amending diets with plant secondary metabolites could address the mechanistic underpinnings of digestive plasticity, and validate our observations.

## Methods

### Sources and rearing conditions of *A. glabripennis* larvae

We obtained *Anoplophora glabripennis* from a laboratory colony maintained at The Pennsylvania State University (PSU) (University Park, PA, USA) under quarantine. The colony has been maintained for 16 generations with regular introgression from *A. glabripennis* populations from infestations in New York, Ohio, and Massachusetts.

We administered one of two diet treatments to the larvae, artificial diet or sugar maple trees (*Acer saccharum*). Since *A. glabripennis* will only oviposit into wood, for larvae fed on artificial diet we collected larvae from maple bolts and transferred them to their respective diet. Briefly, we used mating pairs of *A. glabripennis* held in large, glass mating jars with freshly cut sugar maple twigs for feeding and a small Norway maple (*Acer platanoides*) bolt for oviposition as described previously^42^. About 3 weeks later following the appearance of frass (feces), we removed first instars from these maple bolts and transferred them to a nutrient rich artificial diet^34^, where they fed through the third instar (~60 days). Artificial diet consisted of the following formulation (w v^−1^): 22% cellulose, 9.7% wheat germ, 5.1% tortula yeast, 4.0% agar, 2.9% sucrose, 1.7% casein, 0.8% vitamin mix, 0.5% Wesson salt mix, and 0.2% cholesterol as described in Keena^34^. Oviposition into maple logs and transferring first instars (~3 weeks old) into artificial diet for completion of larval development is part of our standard insect rearing protocol and it has not been associated with any deleterious impacts on larval fitness^45^,^46^. Larvae were maintained in artificial diet at 24 °C in darkness.

We obtained tree-feeding larvae by allowing five pairs of adult male and female *A. glabripennis* of colony origin to mate and oviposit into potted, greenhouse grown sugar maple trees in large walk-in cages contained in a USDA-approved quarantine greenhouse (PSU, University Park, PA) for a period of one month. We transferred the trees to the greenhouse one month prior to oviposition in June for acclimation to greenhouse conditions; the trees were maintained at 24–27 °C under ambient light until they were harvested in August. We removed third instar larvae from the trees ~60 days after the first appearance of frass, which indicates that the larvae have established and are feeding. We anesthetized, sterilized, and dissected the larvae ventrally. We removed the midguts (Supplemental Fig. 1) and froze them immediately in liquid nitrogen followed by storage at −80 °C. We conducted RNA-Seq analyses on midguts collected from four individuals from each diet.

### Preparation of RNA from ALB midguts for sequencing

We isolated total RNA using the RNA Power Soil Microbiome Isolation Kit (MoBio, Carlsbad, CA, USA) from four individual midguts collected from larvae feeding in sugar maple and four individual midguts from insects feeding in artificial diet. We selected this kit because of its ability to eliminate phenolic compounds and polysaccharides from *A. glabripennis* ingested plant material that often co-extract with RNA, can reduce RNA quality, and interfere with downstream applications, such as library prep for next-generation sequencing. We verified the concentration and integrity of extracted RNA using a NanoDrop spectrophotometer (Thermo-Fisher, Rockford, MD, USA) and Agilent Bioanalyzer RNA Nano Assay (Life Technologies, Carlsbad, CA, USA). We depleted ribosomal RNA using the Ribominus Eukaryotic Kit for RNA-Seq (Life Technologies, Carlsbad, CA). Enriched mRNA was polyA purified; multiplexed Illumina libraries were constructed with the TruSeq RNA Sample Prep Kit (Illumina, San Diego, CA, USA). We pooled eight multiplexed Illumina libraries (four derived from the guts of sugar maple reared insects and four from the guts of insects reared on artificial diet) and sequenced them on a single Illumina HiSeq 2000 lane at the University of Delaware Biotechnology Institute (Newark, DE).

### Sequence processing and initial analysis

The HiSeq run yielded ~13 million 101 nt paired end reads per sample. We trimmed forward reads and used ea-utils (https://expressionanalysis.github.io/ea-utils version 27a4809) to filter with the following parameters: we trimmed bases with quality scores less than 30 and discarded reads with more than three ambiguous bases or a mean quality score below 30 (–q 30, –max-ns 0, and –qual-mean 30). We mapped high quality reads of at least 75 nt after quality trimming to the *A. glabripennis* reference genome assembly using Tophat v. 2.0^47^ using default parameters. We used HTSeq v. 0.6.1p^48^ to sum read counts mapped to each locus (gene set: v. 0.5.3 publically available at ftp://ftp.hgsc.bcm.edu/I5K-pilot/Asian_long-horned_beetle/maker_annotation/version_0.5.3/). We then summed reads that spanned multiple features using the union mode (-m union), and discarded reads that did not map uniquely to a single region of the genome (alignment_not_unique).

We performed differential expression analysis with edgeR^49^ (version 3.14.0). Because *A. glabripennis* gut biochemistry and physiology is not well-characterized compared to other insects, we implemented a conservative approach to defining expressed genes in order to reduce false positives. We removed features with fewer than 10 mapped reads, normalized read counts with quantile normalization, and estimated variances using tagwise dispersions. We flagged features as differentially expressed if there was a log fold change greater than ±1.25 and a Benjamini and Hochberg’s FDR (False Discovery Rate) adjusted P-value ≤ 0.05 using a Fisher’s exact test. We used a false discovery rate of 0.05 for this experiment. We used normalized count data from genes that were differentially expressed between the two treatments to perform MDS analysis in R v. 3.1^50^ with the metaMDS function from the ‘vegan’ package^51^.

### Gene ontology enrichment analysis

We performed gene ontology (GO) enrichment analysis using the Bioconductor package GoSeq^52^ (version 1.24.0). We assigned protein coding genes from the *A. glabripennis* genome to GO terms with InterProScan^53^ 5 (version 5.19–58.0) using the –goterms flag. We retrieved basal terms for all GO assignments with the extract_GO_assignments_from_Trinotate_xls.pl script included in the Trinotate package version r20140708 (https://trinotate.github.io/). We used genes that were identified as upregulated in midguts collected from sugar maple-reared larvae to identify GO terms that were enriched in insects feeding in trees. Likewise, we analyzed genes that were upregulated in the midguts of artificial diet-fed larvae to identify GO terms enriched in the diet treatment. In both comparisons, we used the entire list of genes with detectable expression levels (>10 mapped reads) in the midguts of insects feeding in either treatment as a reference to determine enrichment. We weighted all genes by gene length and removed nodes with less than 5 mapped terms from the analysis. We identified enriched GO terms using the Wallenius approximation (“pwf” option) from the molecular function, cellular component, and biological process categories with Benjamini-Hochberg adjusted p-values ≤ 0.05.

### Hierarchical Clustering Analysis

We subjected genes identified as differentially expressed to hierarchical cluster analysis in order to identify groups with correlated expression patterns. RPKM values^54^ for each differentially expressed gene were calculated using the ‘rpkm’ function in edgeR (version 3.14.0) to ensure that differences in gene lengths were not contributing to the clustering patterns. We log_2_ transformed RPKM values and computed Euclidean dissimilarity matrix in R v. 3.1^50^,^51^. We performed hierarchical clustering at the gene-level using the complete linkage method and generated corresponding heatmaps using the heatmap2 command from the gplots package (version 3.0.1).

### KOG and KEGG Pathway Assignment

We assigned *A. glabripennis* genes to KOG (eukaryotic clusters of orthologous gene) categories via an RPS-BLAST^55^ (included with blast version 2.2.23) comparison to the KOG database (accessed 04/01/15)^56^. We used the top scoring blast match with an e-value ≤ 0.00001 for KOG assignments. We also assigned protein coding genes in the *A. glabripennis* genome to KEGG pathways with the KAAS server^57^ accessed 04/15/16 using the bidirectional best hit (BBH) method.

### Availability of sequencing data

The raw Illumina reads used in this study have been deposited into NCBI’s Sequence Read Archive (SRA) and are associated with Bioproject PRJNA279780. The read counts used to compute differential expression have been deposited in Gene Expression Omnibus (GEO) under accession GSE68149. More information regarding sequencing metrics and barcodes used are presented in Supplemental Table 5.

## Additional Information

**How to cite this article**: Mason, C. J. *et al*. Contrasting diets reveal metabolic plasticity in the tree-killing beetle, *Anoplophora glabripennis* (Cerambycidae: Lamiinae). *Sci. Rep.* **6**, 33813; doi: 10.1038/srep33813 (2016).

## Supplementary Material

Supplementary Information

Supplemental Data 1

Supplemental Data 2
