# Supplementary material for: Contrasting diets reveal metabolic plasticity in the tree-killing beetle, Anoplophora glabripennis (Cerambycidae: Lamiinae)
Source: Sci Rep. 2016 Sep 22;6:33813. doi: 10.1038/srep33813 (PMC5031968; doi:10.1038/srep33813)
Supplement: Supplementary Information [file srep33813-s1.doc]

Title: Contrasting diets reveal metabolic plasticity in the tree-killing beetle, *Anoplophora glabripennis* (Cerambycidae: Lamiinae)

**Authors: Charles J. Mason^1^**^ϯ^**, Erin D. Scully^2^**^ϯ*^**, Scott M. Geib^3^, and Kelli Hoover^1*^**

^1^ Department of Entomology and Center for Chemical Ecology, The Pennsylvania State University, University Park, PA 16802 USA

^2^Stored Product Insect and Engineering Research Unit, USDA, Agricultural Research Service, Center for Grain and Animal Health Research, Manhattan, KS 66502 USA

^3^Tropical Crop and Commodity Protection Research Unit, USDA, Agricultural Research Service, Daniel K. Inouye Pacific Basin Agricultural Research Center, Hilo, HI 96720 USA

^*^Address correspondence

^ϯ^These authors contributed equally.

**Supplemental Data Legends:**

**Supplemental Data 1 (Excel file). EdgeR differential expression results.** Genes differentially expressed in the guts of *A. glabripennis* feeding in sugar maple compared to artificial diet were identified using the R package ‘edgeR.’ Variances were computed using tagwise dispersions and differential expression analysis was performed using the exact test function. Log-fold changes and FDR corrected P-values are provided.

**Supplemental Data 2 (Excel file). Identification of genes contributing to variability in expression profiles in the guts of insects reared on sugar maples.** The top 100 genes contributing to differences in expression profiles of insects reared in sugar maple along MDS-2 in Figure 1B were determined using the R package simper.

**Supplemental Tables:**

**Supplemental Table 1. Gene ontology enrichment analysis of insects feeding in sugar maple**. Gene ontology categories that were enriched in *A. glabripennis* larvae feeding in sugar maple compared to those feeding in artificial diet were identified using GoSeq. Categories were eliminated from the analysis if fewer than five genes were assigned and GO categories were identified as being enriched in these insects if they had an FDR-corrected p-value ≤0.05.

| **Category** | **p-value** | **Total Number of Upregulated Genes in Sugar maple** | **Total Number of Genes in Category** | **Term** |
| --- | --- | --- | --- | --- |
| **Biological Process** |  |  |  |  |
| GO:1901605 | 9.72E-003 | 2 | 2 | alpha-amino acid metabolic process |
| GO:0015991 | 9.72E-003 | 2 | 2 | ATP hydrolysis coupled proton transport |
| GO:0005975 | 2.39E-008 | 28 | 91 | carbohydrate metabolic process |
| GO:0019752 | 1.20E-002 | 5 | 15 | carboxylic acid metabolic process |
| GO:0006520 | 1.76E-002 | 4 | 11 | cellular amino acid metabolic process |
| GO:0042180 | 3.55E-003 | 3 | 4 | cellular ketone metabolic process |
| GO:0034645 | 1.53E-003 | 5 | 10 | cellular macromolecule biosynthetic process |
| GO:0015988 | 9.72E-003 | 2 | 2 | energy coupled proton transmembrane transport, against electrochemical gradient |
| GO:0042181 | 3.55E-003 | 3 | 4 | ketone biosynthetic process |
| GO:0009059 | 1.60E-002 | 5 | 16 | macromolecule biosynthetic process |
| GO:0043170 | 1.94E-002 | 48 | 361 | macromolecule metabolic process |
| GO:0008152 | 2.71E-011 | 138 | 816 | metabolic process |
| GO:0006397 | 3.67E-002 | 3 | 8 | mRNA processing |
| GO:0006082 | 1.20E-002 | 5 | 15 | organic acid metabolic process |
| GO:0071704 | 1.09E-008 | 96 | 556 | organic substance metabolic process |
| GO:0006733 | 3.55E-003 | 3 | 4 | oxidoreduction coenzyme metabolic process |
| GO:0043436 | 1.20E-002 | 5 | 15 | oxoacid metabolic process |
| GO:0044238 | 5.24E-008 | 91 | 535 | primary metabolic process |
| GO:0019538 | 1.49E-004 | 37 | 202 | protein metabolic process |
| GO:0006508 | 7.55E-006 | 29 | 124 | proteolysis |
| GO:1901663 | 3.55E-003 | 3 | 4 | quinone biosynthetic process |
| GO:1901661 | 3.55E-003 | 3 | 4 | quinone metabolic process |
| GO:0072593 | 1.20E-003 | 4 | 6 | reactive oxygen species metabolic process |
| GO:0032774 | 7.90E-003 | 4 | 9 | RNA biosynthetic process |
| GO:0044283 | 2.58E-003 | 4 | 7 | small molecule biosynthetic process |
| GO:0044281 | 2.95E-003 | 15 | 70 | small molecule metabolic process |
| GO:0006801 | 1.20E-003 | 4 | 6 | superoxide metabolic process |
| GO:0006351 | 4.34E-004 | 4 | 5 | transcription, DNA-templated |
| GO:0006744 | 9.57E-004 | 3 | 3 | ubiquinone biosynthetic process |
| GO:0006743 | 9.57E-004 | 3 | 3 | ubiquinone metabolic process |
| **Cellular Component** |  |  |  |  |
| GO:1902494 | 1.09E-002 | 7 | 26 | catalytic complex |
| GO:0044464 | 1.02E-004 | 65 | 415 | cell part |
| GO:0044444 | 7.63E-008 | 27 | 90 | cytoplasmic part |
| GO:0005576 | 2.62E-004 | 19 | 81 | extracellular region |
| GO:0044446 | 5.62E-005 | 26 | 117 | intracellular organelle part |
| GO:0044424 | 6.83E-005 | 58 | 354 | intracellular part |
| GO:0032991 | 9.75E-005 | 25 | 114 | macromolecular complex |
| GO:0031966 | 8.66E-003 | 5 | 14 | mitochondrial membrane |
| GO:0044455 | 2.06E-005 | 7 | 11 | mitochondrial membrane part |
| GO:0044429 | 1.87E-006 | 13 | 30 | mitochondrial part |
| GO:0000276 | 5.09E-005 | 5 | 6 | mitochondrial proton-transporting ATP synthase complex, coupling factor F(o) |
| GO:0005739 | 1.20E-003 | 4 | 6 | mitochondrion |
| GO:0031090 | 3.74E-002 | 6 | 26 | organelle membrane |
| GO:0044422 | 5.62E-005 | 26 | 117 | organelle part |
| GO:1990204 | 9.72E-003 | 2 | 2 | oxidoreductase complex |
| GO:0043234 | 4.92E-004 | 22 | 105 | protein complex |
| GO:0045263 | 5.09E-005 | 5 | 6 | proton-transporting ATP synthase complex, coupling factor F(o) |
| GO:0033177 | 5.09E-005 | 5 | 6 | proton-transporting two-sector ATPase complex, proton-transporting domain |
| GO:0005787 | 2.72E-002 | 2 | 3 | signal peptidase complex |
| GO:1990234 | 8.67E-003 | 5 | 14 | transferase complex |
| GO:0000151 | 3.67E-002 | 3 | 8 | ubiquitin ligase complex |
| **Molecular Function** |  |  |  |  |
| GO:0003993 | 1.47E-004 | 10 | 27 | acid phosphatase activity |
| GO:0004017 | 9.57E-004 | 3 | 3 | adenylate kinase activity |
| GO:0016209 | 2.11E-002 | 6 | 23 | antioxidant activity |
| GO:1901505 | 4.09E-002 | 5 | 20 | carbohydrate derivative transporter activity |
| GO:0016840 | 2.73E-002 | 2 | 3 | carbon-nitrogen lyase activity |
| GO:0004180 | 3.13E-002 | 6 | 25 | carboxypeptidase activity |
| GO:0003824 | 2.81E-027 | 347 | 2104 | catalytic activity |
| GO:0008324 | 3.49E-002 | 18 | 116 | cation transmembrane transporter activity |
| GO:0004129 | 3.16E-005 | 9 | 19 | cytochrome-c oxidase activity |
| GO:0009055 | 4.51E-006 | 13 | 32 | electron carrier activity |
| GO:0004175 | 6.51E-007 | 52 | 264 | endopeptidase activity |
| GO:0004348 | 1.35E-004 | 6 | 10 | glucosylceramidase activity |
| GO:0015002 | 3.16E-005 | 9 | 19 | heme-copper terminal oxidase activity |
| GO:0015078 | 5.24E-006 | 14 | 37 | hydrogen ion transmembrane transporter activity |
| GO:0016787 | 2.02E-022 | 165 | 790 | hydrolase activity |
| GO:0016788 | 7.63E-003 | 20 | 114 | hydrolase activity, acting on ester bonds |
| GO:0016798 | 4.97E-027 | 63 | 142 | hydrolase activity, acting on glycosyl bonds |
| GO:0004553 | 3.05E-027 | 63 | 141 | hydrolase activity, hydrolyzing O-glycosyl compounds |
| GO:0005506 | 6.51E-004 | 23 | 114 | iron ion binding |
| GO:0016829 | 1.66E-002 | 7 | 28 | lyase activity |
| GO:0003796 | 9.72E-003 | 2 | 2 | lysozyme activity |
| GO:0050136 | 8.21E-003 | 3 | 5 | NADH dehydrogenase (quinone) activity |
| GO:0008137 | 8.21E-003 | 3 | 5 | NADH dehydrogenase (ubiquinone) activity |
| GO:0003954 | 1.64E-004 | 5 | 7 | NADH dehydrogenase activity |
| GO:0097659 | 4.34E-004 | 4 | 5 | nucleic acid-templated transcription |
| GO:0019205 | 8.46E-004 | 6 | 13 | nucleobase-containing compound kinase activity |
| GO:0015932 | 4.09E-002 | 5 | 20 | nucleobase-containing compound transmembrane transporter activity |
| GO:0005337 | 3.33E-002 | 5 | 19 | nucleoside transmembrane transporter activity |
| GO:0019201 | 4.34E-004 | 4 | 5 | nucleotide kinase activity |
| GO:0016491 | 6.13E-013 | 74 | 318 | oxidoreductase activity |
| GO:0016675 | 3.16E-005 | 9 | 19 | oxidoreductase activity, acting on a heme group of donors |
| GO:0016676 | 3.16E-005 | 9 | 19 | oxidoreductase activity, acting on a heme group of donors, oxygen as acceptor |
| GO:0016679 | 2.72E-002 | 2 | 3 | oxidoreductase activity, acting on diphenols and related substances as donors |
| GO:0016681 | 2.72E-002 | 2 | 3 | oxidoreductase activity, acting on diphenols and related substances as donors, cytochrome as acceptor |
| GO:0016651 | 1.64E-004 | 7 | 14 | oxidoreductase activity, acting on NAD(P)H |
| GO:0016655 | 8.21E-003 | 3 | 5 | oxidoreductase activity, acting on NAD(P)H, quinone or similar compound as acceptor |
| GO:0008233 | 2.43E-006 | 61 | 341 | peptidase activity |
| GO:0070011 | 2.21E-006 | 61 | 340 | peptidase activity, acting on L-amino acid peptides |
| GO:0016791 | 2.38E-004 | 14 | 50 | phosphatase activity |
| GO:0042578 | 1.06E-003 | 16 | 70 | phosphoric ester hydrolase activity |
| GO:0016776 | 2.71E-004 | 6 | 11 | phosphotransferase activity, phosphate group as acceptor |
| GO:0004650 | 1.00E-009 | 12 | 16 | polygalacturonase activity |
| GO:0000988 | 1.35E-002 | 6 | 21 | protein binding transcription factor activity |
| GO:0046982 | 2.44E-002 | 4 | 12 | protein heterodimerization activity |
| GO:0001104 | 1.60E-002 | 5 | 16 | RNA polymerase II transcription cofactor activity |
| GO:0001076 | 1.60E-002 | 5 | 16 | RNA polymerase II transcription factor binding transcription factor activity |
| GO:0017171 | 7.82E-008 | 39 | 161 | serine hydrolase activity |
| GO:0004252 | 6.80E-008 | 38 | 154 | serine-type endopeptidase activity |
| GO:0008236 | 7.82E-008 | 39 | 161 | serine-type peptidase activity |
| GO:0003735 | 4.06E-050 | 72 | 101 | structural constituent of ribosome |
| GO:0005198 | 3.24E-019 | 76 | 259 | structural molecule activity |
| GO:0004298 | 1.64E-004 | 5 | 7 | threonine-type endopeptidase activity |
| GO:0070003 | 1.64E-004 | 5 | 7 | threonine-type peptidase activity |
| GO:0003712 | 1.35E-002 | 6 | 21 | transcription cofactor activity |
| GO:0000989 | 1.35E-002 | 6 | 21 | transcription factor binding transcription factor activity |
| GO:0046914 | 3.98E-002 | 33 | 245 | transition metal ion binding |
| GO:0008121 | 2.72E-002 | 2 | 3 | ubiquinol-cytochrome-c reductase activity |

**Supplemental Table 2. Gene ontology enrichment analysis of insects feeding in artificial diet**. Gene ontology categories that were enriched in *A. glabripennis* larvae feeding in artificial diet compared to those feeding in sugar maple were identified using GoSeq. Categories were eliminated from the analysis if fewer than five genes were assigned and GO categories were identified as being enriched in these insects if they had an FDR-corrected p-value ≤0.05.

| **Category** | **p-value** | **Total Number of Downregulated Genes in sugar maple** | **Total Number of Genes in Category** | **Term** |
| --- | --- | --- | --- | --- |
| **Biological Process** |  |  |  |  |
| GO:0065007 | 1.54E-003 | 6 | 170 | biological regulation |
| GO:0006259 | 3.27E-003 | 2 | 95 | DNA metabolic process |
| GO:0050789 | 7.07E-003 | 6 | 148 | regulation of biological process |
| GO:0050794 | 7.07E-003 | 6 | 148 | regulation of cellular process |
| GO:0015074 | 1.73E-002 | 2 | 75 | DNA integration |
| GO:0051179 | 2.77E-002 | 12 | 205 | localization |
| GO:0006810 | 3.37E-002 | 12 | 201 | transport |
| GO:0051234 | 3.37E-002 | 12 | 201 | establishment of localization |
| GO:0007165 | 3.51E-002 | 3 | 81 | signal transduction |
| **Molecular Function** |  |  |  |  |
| GO:0003676 | 0.00E+000 | 71 | 1989 | nucleic acid binding |
| GO:0005488 | 0.00E+000 | 280 | 5318 | binding |
| GO:0097159 | 0.00E+000 | 92 | 2421 | organic cyclic compound binding |
| GO:1901363 | 0.00E+000 | 92 | 2419 | heterocyclic compound binding |
| GO:0005515 | 0.00E+000 | 104 | 1873 | protein binding |
| GO:0003677 | 0.00E+000 | 17 | 612 | DNA binding |
| GO:0003674 | 1.99E-009 | 756 | 8439 | molecular_function |
| GO:0060089 | 3.61E-006 | 4 | 211 | molecular transducer activity |
| GO:0017076 | 3.81E-006 | 8 | 282 | purine nucleotide binding |
| GO:0001882 | 6.58E-006 | 8 | 275 | nucleoside binding |
| GO:0001883 | 6.58E-006 | 8 | 275 | purine nucleoside binding |
| GO:0032549 | 6.58E-006 | 8 | 275 | ribonucleoside binding |
| GO:0032550 | 6.58E-006 | 8 | 275 | purine ribonucleoside binding |
| GO:0032555 | 6.58E-006 | 8 | 275 | purine ribonucleotide binding |
| GO:0035639 | 6.58E-006 | 8 | 275 | purine ribonucleoside triphosphate binding |
| GO:0004871 | 1.45E-005 | 4 | 195 | signal transducer activity |
| GO:0004888 | 1.70E-005 | 3 | 174 | transmembrane signaling receptor activity |
| GO:0004872 | 2.04E-005 | 4 | 191 | receptor activity |
| GO:0043168 | 2.05E-005 | 11 | 308 | anion binding |
| GO:0036094 | 3.70E-005 | 19 | 417 | small molecule binding |
| GO:0000166 | 4.19E-005 | 19 | 415 | nucleotide binding |
| GO:1901265 | 4.19E-005 | 19 | 415 | nucleoside phosphate binding |
| GO:0032553 | 4.99E-005 | 10 | 280 | ribonucleotide binding |
| GO:0097367 | 4.99E-005 | 10 | 280 | carbohydrate derivative binding |
| GO:0038023 | 7.87E-005 | 4 | 175 | signaling receptor activity |
| GO:0042302 | 7.88E-005 | 2 | 137 | structural constituent of cuticle |
| GO:0030554 | 1.10E-004 | 4 | 171 | adenyl nucleotide binding |
| GO:0005524 | 1.95E-004 | 4 | 164 | ATP binding |
| GO:0032559 | 1.95E-004 | 4 | 164 | adenyl ribonucleotide binding |
| GO:0043167 | 1.96E-004 | 74 | 1075 | ion binding |
| GO:0004672 | 2.50E-004 | 4 | 161 | protein kinase activity |
| GO:0004930 | 4.71E-004 | 1 | 97 | G-protein coupled receptor activity |
| GO:0016773 | 5.82E-004 | 7 | 199 | phosphotransferase activity, alcohol group as acceptor |
| GO:0046983 | 4.72E-003 | 5 | 139 | protein dimerization activity |
| GO:0005216 | 7.13E-003 | 4 | 118 | ion channel activity |
| GO:0015267 | 7.13E-003 | 4 | 118 | channel activity |
| GO:0022803 | 7.13E-003 | 4 | 118 | passive transmembrane transporter activity |
| GO:0022838 | 7.13E-003 | 4 | 118 | substrate-specific channel activity |
| GO:0003777 | 7.50E-003 | 0 | 47 | microtubule motor activity |
| GO:0001071 | 1.04E-002 | 9 | 184 | nucleic acid binding transcription factor activity |
| GO:0003700 | 1.04E-002 | 9 | 184 | sequence-specific DNA binding transcription factor activity |
| GO:0003723 | 1.11E-002 | 21 | 337 | RNA binding |
| GO:0060589 | 1.14E-002 | 0 | 43 | nucleoside-triphosphatase regulator activity |
| GO:0005525 | 1.19E-002 | 4 | 111 | GTP binding |
| GO:0019001 | 1.19E-002 | 4 | 111 | guanyl nucleotide binding |
| GO:0032561 | 1.19E-002 | 4 | 111 | guanyl ribonucleotide binding |
| GO:0030695 | 1.40E-002 | 0 | 41 | GTPase regulator activity |
| GO:0016817 | 1.57E-002 | 4 | 107 | hydrolase activity, acting on acid anhydrides |
| GO:0016818 | 1.69E-002 | 4 | 106 | hydrolase activity, acting on acid anhydrides, in phosphorus-containing anhydrides |
| GO:0016462 | 1.94E-002 | 4 | 104 | pyrophosphatase activity |
| GO:0017111 | 1.94E-002 | 4 | 104 | nucleoside-triphosphatase activity |
| GO:0005096 | 2.13E-002 | 0 | 37 | GTPase activator activity |
| GO:0016301 | 2.51E-002 | 12 | 207 | kinase activity |
| GO:0005083 | 2.62E-002 | 0 | 35 | small GTPase regulator activity |
| GO:0016740 | 2.63E-002 | 41 | 551 | transferase activity |
| GO:0022836 | 3.02E-002 | 2 | 68 | gated channel activity |
| GO:0005085 | 3.23E-002 | 0 | 33 | guanyl-nucleotide exchange factor activity |
| GO:0004984 | 3.58E-002 | 0 | 32 | olfactory receptor activity |
| GO:0004842 | 3.98E-002 | 0 | 31 | ubiquitin-protein transferase activity |
| GO:0019787 | 3.98E-002 | 0 | 31 | small conjugating protein ligase activity |
| GO:0003774 | 4.13E-002 | 2 | 64 | motor activity |

**Supplemental Table 3. Detoxification genes downregulated in insects feeding in sugar maple.** Several glutathione S-transferases, UDP-glucuronsyl transferases, cytochrome P450s, and carboxylesterases were downregulated in insets feeding in sugar maple. Log fold change and FDR corrected p-values are presented.

| **Gene ID** | **Log Fold Change** | **FDR corrected p-value** | **Gene Annotation** |
| --- | --- | --- | --- |
| AGLA006314 | -3.70 | 1.03E-04 | Carboxylesterase |
| AGLA012201 | -3.39 | 1.85E-05 | Carboxylesterase |
| AGLA013180 | -3.37 | 4.73E-10 | Carboxylesterase |
| AGLA015450 | -2.46 | 2.25E-03 | Carboxylesterase |
| AGLA015448 | -2.16 | 8.00E-03 | Carboxylesterase |
| AGLA017069 | -2.03 | 4.58E-06 | Carboxylesterase |
| AGLA021921 | -1.92 | 1.09E-02 | Carboxylesterase |
| AGLA017068 | -1.89 | 3.27E-03 | Carboxylesterase |
| AGLA013189 | -1.76 | 3.65E-03 | Carboxylesterase |
| AGLA013178 | -1.65 | 1.66E-03 | Carboxylesterase |
| AGLA015447 | -1.40 | 3.38E-03 | Carboxylesterase |
| AGLA003185 | -2.24 | 3.06E-02 | UDP-glucuronsyl transferase |
| AGLA003186 | -2.24 | 7.85E-03 | UDP-glucuronsyl transferase |
| AGLA006649 | -1.71 | 2.14E-02 | UDP-glucuronsyl transferase |
| AGLA010722 | -1.61 | 5.40E-02 | UDP-glucuronsyl transferase |
| AGLA004615 | -1.47 | 1.41E-01 | UDP-glucuronsyl transferase |
| AGLA009328 | -1.37 | 1.03E-01 | UDP-glucuronsyl transferase |
| AGLA004762 | -1.26 | 3.75E-03 | UDP-glucuronsyl transferase |
| AGLA011087 | -3.64 | 7.85E-09 | Cytochrome P450 |
| AGLA007075 | -3.04 | 5.16E-04 | Cytochrome P450 |
| AGLA011691 | -2.83 | 9.72E-06 | Cytochrome P450 |
| AGLA007088 | -2.75 | 5.61E-08 | Cytochrome P450 |
| AGLA007083 | -2.55 | 5.37E-06 | Cytochrome P450 |
| AGLA008947 | -2.32 | 5.76E-03 | Cytochrome P450 |
| AGLA007086 | -2.12 | 3.74E-03 | Cytochrome P450 |
| AGLA006737 | -2.12 | 3.13E-03 | Cytochrome P450 |
| AGLA007103 | -2.08 | 1.26E-03 | Cytochrome P450 |
| AGLA008135 | -2.07 | 7.09E-03 | Cytochrome P450 |
| AGLA001979 | -2.07 | 4.33E-05 | Cytochrome P450 |
| AGLA011689 | -1.71 | 5.87E-03 | Cytochrome P450 |
| AGLA011688 | -1.63 | 2.00E-02 | Cytochrome P450 |
| AGLA001980 | -1.45 | 1.95E-02 | Cytochrome P450 |
| AGLA000009 | -1.42 | 1.36E-02 | Cytochrome P450 |

**Supplemental Table 4. Expression patterns of heatshock proteins in the guts of larvae feeding on sugar maple and artificial diet.** The expression levels of heatshock proteins were impacted by feeding in different diets. Log-fold change and FDR-corrected p-value are presented. Negative log fold changes indicate downregulation of genes in sugar maple compared to artificial diet, while positive values indicate upregulation.

| **Gene ID** | **Annotation** | **Log Fold Change** | **FDR Corrected p-value** |
| --- | --- | --- | --- |
| **AGLA003672** | **Heat Shock protein 70 family** | **-5.34** | **3.43E-14** |
| **AGLA003513** | **Heat Shock protein 70 family** | **-2.82** | **7.58E-04** |
| **AGLA008048** | **Heat Shock protein 70 family** | **-2.77** | **2.82E-06** |
| **AGLA013758** | **Heat Shock protein 70 family** | **-1.98** | **7.21E-03** |
| **AGLA014761** | **Heat Shock protein 90 family** | **-1.39** | **1.66E-02** |
| **AGLA001322** | **Heat Shock protein 70 family** | **-1.29** | **2.18E-02** |
| AGLA003161 | Heat Shock protein 90 family | -1.09 | 1.20E-01 |
| AGLA007615 | Heat shock chaperonin-binding | -0.97 | 6.67E-02 |
| AGLA004440 | Heat Shock protein 90 family | -0.51 | 3.94E-01 |
| AGLA003783 | Heat Shock protein 70 family | -0.38 | 4.23E-01 |
| AGLA010928 | Heat Shock protein 20 family | -0.19 | 6.96E-01 |
| AGLA003512 | Heat Shock protein 70 family | -0.15 | 8.06E-01 |
| AGLA003003 | Heat Shock protein 70 family | -0.07 | 9.89E-01 |
| AGLA010780 | Heat Shock protein 70 family | -0.06 | 9.16E-01 |
| AGLA005216 | Heat Shock protein 70 family | -0.05 | 9.66E-01 |
| AGLA009858 | Heat Shock protein 70 family | 0.00 | 1.00E+00 |
| AGLA016266 | Heat Shock protein 70 family | 0.12 | 8.66E-01 |
| AGLA002692 | Heat Shock protein 20 family | 0.33 | 4.90E-01 |
| AGLA001900 | Heat Shock protein 40 family | 0.34 | 5.16E-01 |
| AGLA010527 | Heat Shock protein 20 family | 0.46 | 4.54E-01 |
| AGLA010923 | Heat Shock protein 40 family | 0.56 | 3.21E-01 |
| **AGLA012808** | **Heat Shock protein 20 family** | **1.11** | **2.18E-02** |
| **AGLA012339** | **Heat Shock protein 70 family** | **1.13** | **9.11E-03** |
| **AGLA001902** | **Heat Shock protein 20 family** | **1.26** | **3.59E-03** |
| **AGLA010927** | **Heat Shock protein 20 family** | **1.27** | **2.14E-02** |
| **AGLA006890** | **Heat Shock protein 20 family** | **1.46** | **2.36E-03** |
| AGLA005022 | Heat Shock protein 70 family | 1.51 | 1.22E-01 |
| AGLA018259 | Heat Shock protein 20 family | 1.63 | 2.08E-01 |
| **AGLA000750** | **Heat Shock protein 20 family** | **1.65** | **2.39E-02** |
| **AGLA006012** | **Heat Shock protein 20 family** | **1.79** | **5.66E-03** |
| **AGLA010926** | **Heat Shock protein 20 family** | **2.17** | **2.05E-03** |
| **AGLA010057** | **Heat Shock protein 20 family** | **3.21** | **1.34E-06** |
| **AGLA010526** | **Heat Shock protein 20 family** | **3.39** | **6.00E-05** |
| AGLA001662 | Heat Shock protein 70 family | N/A | N/A |
| AGLA001831 | Heat Shock protein 20 family | N/A | N/A |
| AGLA006375 | Heat shock factor (HSF)-type, DNA-binding | N/A | N/A |
| AGLA009043 | Heat Shock protein 70 family | N/A | N/A |
| AGLA009425 | Heat Shock protein 20 family | N/A | N/A |
| AGLA010525 | Heat Shock protein 20 family | N/A | NA |
| AGLA011750 | Heat Shock protein 40 family | N/A | NA |

**Supplemental Table 5. Illumina sequencing metrics.** Library yields and mapping information from RNA-Seq libraries prepared from the midguts of four diet- and sugar maple-fed *A. glabripennis* larvae. Libraries were sequenced on the Illumina HiSeq 2000 platform and were mapped to the reference genome using Tophat 2.0. Reads that mapped to multiple features were not counted.

| Sample Name | Barcode | # Reads Obtained from Sequencing (Gb) | # Reads Retained after Quality Filtering (Gb) | # Reads Uniquely Mapped to Reference Genome | SRA Run Number |
| --- | --- | --- | --- | --- | --- |
| ALB diet fed 1 | CGATGT | 14,386,396 (1.52 Gb) | 13,467,025 (1.40 Gb) | 8,438,046 | SRR2000521 |
| ALB diet fed 2 | TGACCA | 14,365,044 (1.52 Gb) | 13,490,262 (1.40 Gb) | 8,342,467 | SRR2000536 |
| ALB diet fed 3 | ATCACG | 11,229,507  (1.28 Gb) | 11,229,507 (1.17 Gb) | 10,016,025 | SRR2000537 |
| ALB diet fed 4 | TTAGGC | 11,408,020 (1.21 Gb) | 10,672,394 (1.11 Gb) | 6,565,424 | SRR2000538 |
| ALB sugar maple fed 1 | AGTTCC | 13,691,666 (1.45 Gb) | 12,862,413 (1.33 Gb) | 7,002,355 | SRR2000539 |
| ALB sugar maple fed 2 | ATGTCA | 10,690,300 (1.13 Gb) | 9,463,842 (0.98 Gb) | 4,071,121 | SRR2000540 |
| ALB sugar maple fed 3 | CCGTCC | 7,135,992  (0.76 Gb) | 6,775,648 (0.70 Gb) | 3,569,873 | SRR2000541 |
| ALB sugar maple fed 4 | GTCCGC | 14,018,281  (1.49 Gb) | 13,252,190 (1.37 Gb) | 6,805,480 | SRR2000542 |

**Supplemental Figures:**

**Supplemental Fig 1. Gut of *Anoplophora glabripennis*.** The midgut is a long and serpentine tube that forms two loops (denoted by *). The anterior region of the gut is often dark orange to brown in color, and the anterior regions are pale yellow to brown. The hindgut is very small compared to the midgut, about 5% of the length of the midgut. The foregut is not shown.

**Supplemental Figure 2. RPKM values of digestive proteinases more highly expressed in diet-reared larvae.** RPKM levels were computed using the ‘rpkm’ function in edgeR. * p<0.05 **p<0.001 *** p<0.0000001. Letters beneath the bargraph represent PFAM domain accessions. A: IPR001548 Peptidase M2 , peptidyl-dipeptidase A; B: PF13620 Carboxypeptidase regulatory-like domain; C; PF00656 Caspase domain; D: PF03571 Peptidase family; E: PF00675 Insulinase (Peptidase family M16); F: PF00326 Prolyl oligopeptidase family 2; G: PF00079 Serpin (serine protease inhibitor); H: PF00089 Trypsin; I: PF09668 Aspartyl protease.

**
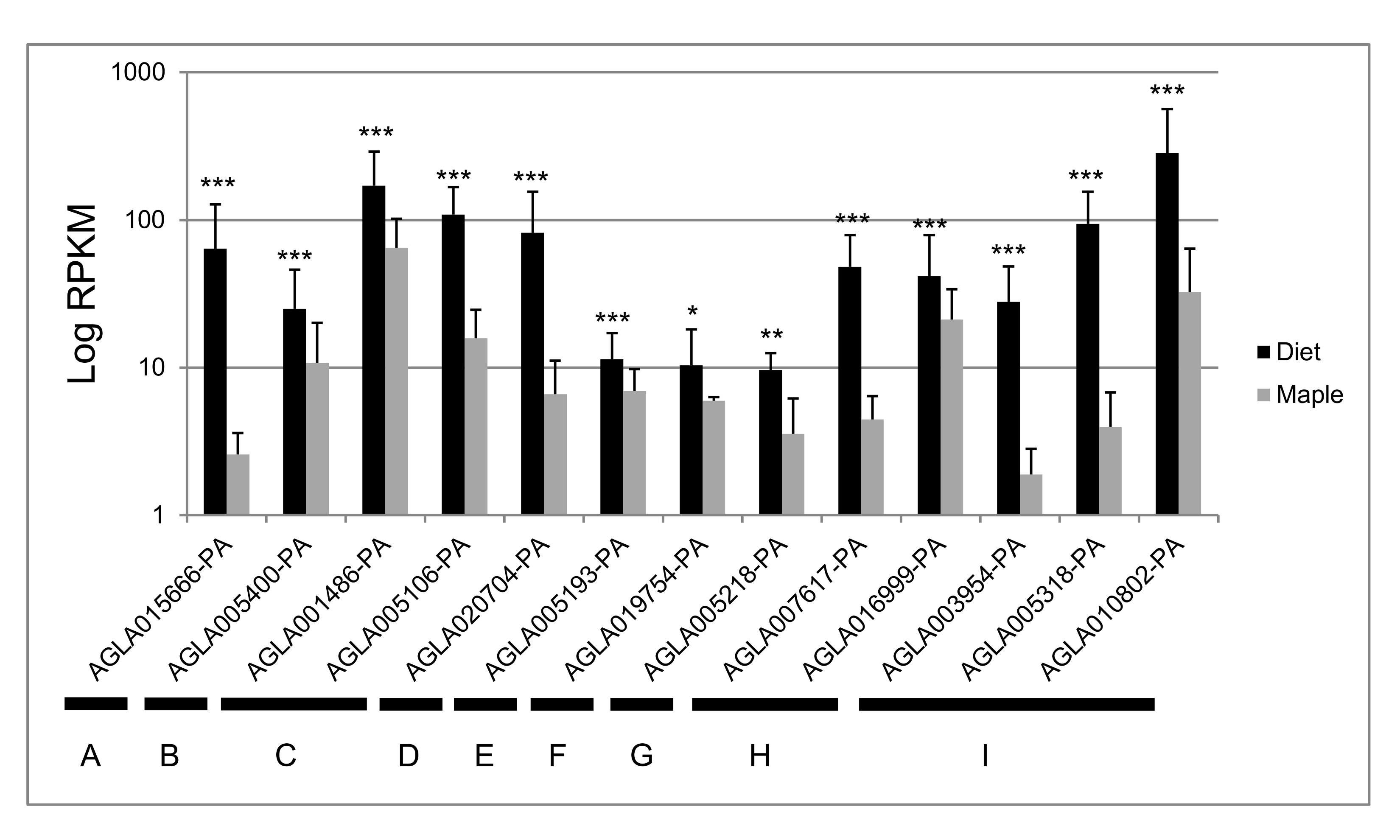
**
